# A major zebrafish polymorphism resource for genetic mapping

**DOI:** 10.1186/gb-2007-8-4-r55

**Published:** 2007-04-11

**Authors:** Kevin M Bradley, J Bradford Elmore, Joan P Breyer, Brian L Yaspan, Jason R Jessen, Ela W Knapik, Jeffrey R Smith

**Affiliations:** 1 Department of Medicine, Vanderbilt University School of Medicine, Garland Avenue, Nashville, TN 37232-0275, USA; 2 Department of Cancer Biology, Vanderbilt University School of Medicine, Garland Avenue, Nashville, TN 37232-0275, USA; 3 Vanderbilt-Ingram Cancer Center, Vanderbilt University School of Medicine, Garland Avenue, Nashville, TN 37232-0275, USA; 4 Medical Research Service, VA Tennessee Valley Healthcare System, Nashville, TN 37212, USA

## Abstract

We have identified 645,088 candidate polymorphisms in zebrafish and observe a single nucleotide polymorphism (SNP) validation rate of 71% to 86%, improving with polymorphism confidence score. Variant sites are non-random, with an excess of specific novel T- and A-rich motifs. We positioned half of the polymorphisms on zebrafish genetic and physical maps as a resource for positional cloning. We further demonstrate bulked segregant analysis using the anchored SNPs as a method for high-throughput genetic mapping in zebrafish.

## Abstract 2

645,088 candidate polymorphisms in zebrafish were identified and positioned on genetic and physical maps as a resource for positional cloning.

## Background

The zebrafish model organism (*Danio rerio*) is increasingly used to genetically map traits relevant to vertebrate development, pathophysiology, and behavior. Forward genetic screens can generate thousands of mutations with phenotypes that are salient to human development and pathophysiology. These generate novel opportunities to delineate the molecular processes underlying important traits. The Zebrafish Information Network (ZFIN) currently annotates 2,744 characterized mutants, many generated in large-scale mutagenesis screens [1]. Most of these remain to be genetically mapped and positionally cloned. Here we describe an extensive single nucleotide polymorphism (SNP) resource of sufficient density anchored to genetic maps to facilitate SNP-based positional cloning in zebrafish.

Mutagenesis screens have been devised to induce and recover mutations in zebrafish on a large scale, in a two-generation breeding scheme [2-5]. A cross of F1 heterozygous carriers of a recessive trait yields mutant progeny that are homozygous at a marker fully linked to the trait locus, and yields wild-type progeny that are either heterozygous or homozygous for the alternative allele at the marker. In contrast, alleles of unlinked markers segregate independently of the trait. Comparison of relative allele dosage between mutant versus wild-type progeny can be efficiently done within DNA pools. The identification of linkage by comparing relative allele frequencies in mutant and wild-type DNA pools is a powerful tool that has been successfully employed in zebrafish genetic screens, termed bulked segregant analysis [6]. Individual progeny may subsequently be genotyped to estimate genetic distance between a linked marker and the trait locus. The potential for rapid and inexpensive genotyping employing SNPs could accelerate gene discovery in large-scale mutagenesis screens as well as in individual experimental crosses, further improving the utility of the zebrafish model organism.

Positional cloning in zebrafish has been facilitated by the development of genetic polymorphism maps. These maps have assigned marker positions based upon either meiotic crosses or radiation hybrid panels. ZFIN serves as the bioinformatics repository for the maps and also hosts an integrated map [1]. In aggregate this resource contains 4,303 simple sequence length polymorphisms (SSLP) and 2,029 SNP markers. Recent data mining of expressed sequence tags (ESTs) yielded an additional set of 51,769 candidate SNPs [7]. Two-thirds of those candidate SNPs were uniquely positioned in the Sanger Institute's Zv5 draft sequence assembly of the roughly 1,700 Mb zebrafish genome. Future completion of the genome assembly and gene annotation will ultimately provide a very powerful resource for positional candidate mapping of zebrafish traits salient to human health and biology. This study expands the marker density of these maps by an order of magnitude with the addition of over a half million new markers (roughly 550K SNPs, 80K insertion-deletions, and 18K SSLPs).

Our study identified candidate SNPs by comparing whole-genome shotgun sequence of a population to reference sequence of finished clones. The principal goal was to identify SNPs of finished bacterial artificial chromosome (BAC) clones that are currently positioned on the extensive genetic maps of zebrafish, thus forming an immediate resource for SNP-based genetic mapping. Each of these BACs is positioned on a genetic map by anchorage of previously mapped markers, which in turn provides the location of many newly identified SNPs within the BAC. The great number of newly discovered SNPs of each BAC increases the likelihood that one at a map position will be informative for a given experimental cross. This facilitates high-throughput genetic mapping across diverse experimental designs and laboratories. In order to demonstrate that the newly discovered SNP maps are effective in positional cloning experiments, we employed them in bulked segregant analysis of the previously cloned *trilobite *locus and identified linkage to its correct genetic position [8]. We additionally identified SNPs of BACs that are not currently anchored to genetic maps but form an extensive resource to improve uniformity of map coverage. Accurate assignment of these BACs to genetic positions, or to physical positions within the future completed zebrafish sequence assembly, will augment the utility of the SNP linkage mapping resource.

## Results

### Prediction and validation of SNPs

We screened finished BAC sequence for evidence of polymorphism in shotgun sequence generated predominantly from the Tuebingen (TU) mapping strain. These BACs represent 39% of the Zv6 draft genome assembly. We deemed 627,185 variants to be candidate polymorphisms, including 548,508 bi-allelic SNPs and 78,327 insertion-deletions, based upon a study-assigned polymorphism confidence score ≥4. The scoring algorithm counts observations of differing alleles and biases against sequencing errors as well as non-binary SNPs, which allows selection of variants with the most compelling evidence of polymorphism (detailed in Materials and methods). We additionally identified 17,903 simple tandem repeats with sequence evidence of length polymorphism (67% dimers, 10% trimers, 19% tetramers, and 4% pentamers). To test the accuracy of SNP identification and to correlate confidence score with experimental validation rate, we randomly selected a set of 375 candidate SNPs among those with a confidence score ≥4 and genotyped a wild-type sample of 40 TU [2] population fish and 2 fish of each of three partially inbred strains (SJD, SJC, and SJA derived from DAR, C32, and AB, respectively) [9]. Among the candidate SNPs, we observed polymorphism for 267 (71%) within the population sample. Despite masking clones for repetitive sequence to preclude false SNP detection, 38 of the 375 assays (10%) appeared to detect non-unique loci where no TU fish were homozygotes. Exclusion of such non-unique loci would have improved the polymorphism rate to 79%. Among polymorphic SNPs, 42% were polymorphic across or within the three partially inbred strains, 53% were polymorphic only within the TU population sample, and 5% were polymorphic only in comparison of a partially inbred strain to the TU population. Detail of allele frequency in these lines is provided in Additional data file 1.

Our study-assigned confidence score correlated well with the likelihood of validation for sampled candidate SNPs. Increasing the confidence score threshold for SNP selection to 5, 6, or 7 improved the validation rate to 80%, 83%, and 86%, respectively. By comparison, we previously observed polymorphism for 77.1% of zebrafish SNPs in dbSNP build 124 (933 of 1,210 for which sufficient unique sequence was available) when evaluated in the partially inbred strains in which the SNPs were originally discovered [10,11] (and unpublished data of JBE). The relative proportions of the possible binary single nucleotide variant sites is stable among candidate SNPs with a confidence score over 4 (51.3% A/G or C/T, 22.9% A/C or G/T, 20.3% A/T, and 5.5% C/G, as detailed in Additional data file 2), and is different in comparison to the corresponding distribution of human SNPs (65.3%, 18.2%, 7.6%, and 8.9%, respectively). As in the human and mouse genomes, the majority of the candidate zebrafish SNPs are C to T, or G to A transitions. However, in zebrafish the proportion of these SNPs is notably less than in human.

We conducted a preliminary analysis of neighboring base context of the candidate zebrafish SNPs within unique finished sequence. The frequency of neighboring bases at candidate zebrafish SNPs is summarized in Tables 1 and 2. A bias of sequence context is apparent at G/T transversion SNPs for sites flanked by ^5'^T-N-T^3'^. Following the individual position analytic approach of Zhao and Boerwinkle [12], a proportion bias for T is present at the nucleotide 5' and 3' of G/T SNPs when normalized against the genome average (Table 2). The converse is apparent for C/A transversion SNPs. These observations contrast with prior observations within the human genome [12]. Among other SNP categories, a bias of sequence context is apparent at C/T, A/G, and A/T SNPs for ^5'^T-N-A^3' ^sites (Table 1). A proportion bias for T is present at the nucleotide 5' to C/T and A/T SNPs (Table 2). A proportion bias for A is present at the nucleotide 3' to A/G and A/T SNPs. However, at C/T transition SNPs the proportion bias for G at the 3' nucleotide position is greater than bias for A. Furthermore, for A/G transition SNPs, the proportion bias for C at the 5' position is most prominent. The direction of the bias, though not the extent, is concordant with Zhao and Boerwinkle's observations within the human genome, interpreted as hypermutability effects of CpG dinucleotides. More comprehensive analyses are warranted with completion of the annotated zebrafish genome.

**Figure 1:**
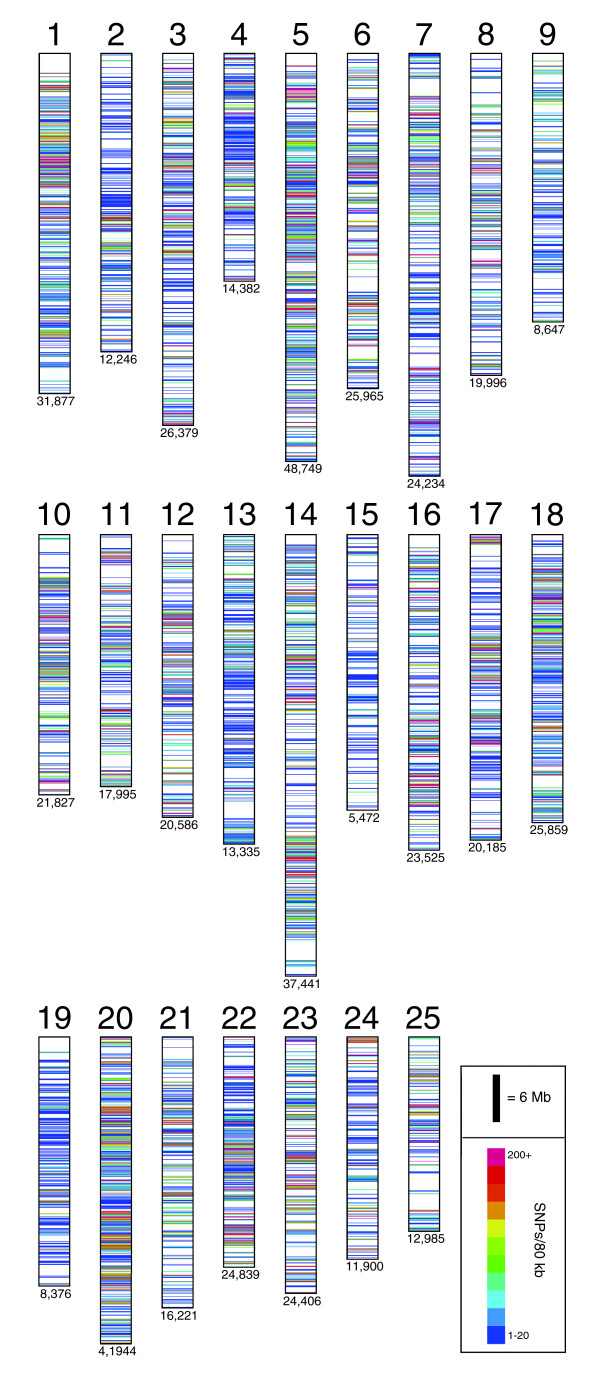
Distribution of SNPs within the Zv6 draft zebrafish genome assembly. SNP density is represented by color, where blank areas indicate absence of SNPs of finished clones. The number of candidate SNPs of confidence score ≥4 is indicated at the bottom of each chromosome. SNPs are distributed among 4,319 BACs (an additional 91 were not included in Zv6) representing 39% of the draft genome.

The overall distribution of SNPs of confidence score ≥4 on the Zv6 draft sequence assembly is presented in Figure 1. The largest gap between candidate SNPs of confidence score ≥4 on the assembly is 4.0 Mb. Under the projection that 71% of candidate variants will validate (391K of those of confidence score ≥4), the mean density is one SNP per 3.9 kb of the 1.55 billion base pair Zv6 assembly, or one SNP per 1.8 kb of finished sequence tested. This calculation represents an observed density that is likely to be an underestimate of the actual SNP density within the predominantly TU strain sequence studied, and does not account for variants yet to be discovered within and across additional strains. Nevertheless, our data suggest that the SNP density and distribution are sufficient to generate a high fidelity linkage mapping set.

### Assignment of genetic map positions

To facilitate use of these novel markers for genetic linkage analysis, we positioned a subset of the polymorphisms on existing genetic maps. This enables reliance upon genetic position for selection of mapping markers, in addition to an increasingly accurate physical position. Four of the highly annotated zebrafish genetic maps include the Heat Shock (HS) meiotic map [13] (3,955 markers), the Boston MGH meiotic map [14] (3,842 markers), the T51 radiation hybrid (RH) map [15] (14,988 markers), and the Loeb/NIH/5000/4000 (LN54) RH map [16] (4,200 markers). To anchor the discovered polymorphisms to these existing genetic maps, we identified BACs containing previously mapped markers. This in turn positioned the newly discovered SNP, SSLP, and insertion-deletion polymorphisms of the identified BACs on the genetic maps. Potential recombination or radiation-induced breakpoints within a BAC region limits precision of these surrogate positions; however, the close physical proximity of markers within each BAC provides a reasonable estimate of map location. The recombinational distance in centiMorgans between a previously annotated marker of a given BAC and its other newly discovered markers remains unknown, but meiotic separation is likely to be limited given the close physical proximity. Better estimates can be made for radiation hybrid maps. One centiRay corresponds to 61 kb on the T51 map and 148 kb on the LN54 map, and the average finished clone is 153 kb in size. Thus, the surrogate position of a SNP on an RH map is estimated to be within approximately 2.5 cR on the T51 map and approximately 1 cR on the LN54 map, relative to a position obtained by direct assay.

**Figure 2:**
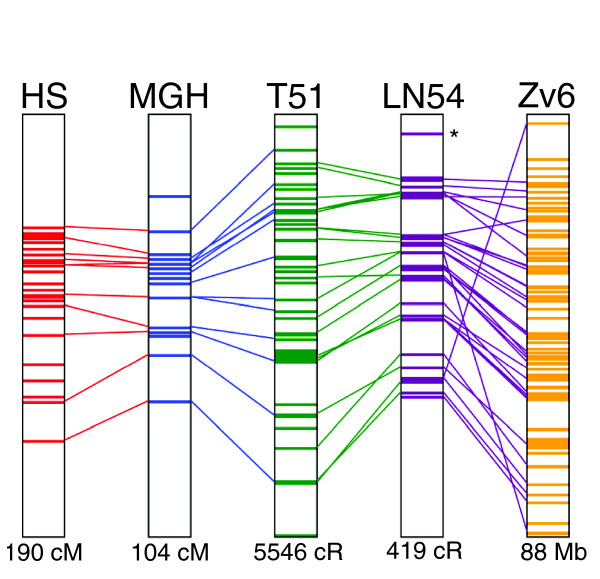
Comparison of positions of SNPs anchored on four genetic maps and on the draft sequence assembly of chromosome 7. Finished clones harboring newly discovered polymorphisms are anchored on a genetic map by content of previously mapped markers. Clone positions on meiotic maps (heat shock (HS) and Boston MGH), radiation hybrid maps (T51 and LN54), and the Zv6 draft physical sequence assembly are provided. Where a clone is common to two adjacent maps, a connecting line indicates corresponding positions. The asterisk indicates the position of BAC zC258D20, linked to the *trilobite *locus.

Typically, a finished BAC was positioned on genetic maps by redundant marker content; the mean number of genetic markers to anchor a finished clone was 4.9. Pairwise comparisons of meiotic and RH maps reveal very good concordance and support their utility for positional cloning. The most discordant among these pairwise comparisons was a 15% marker order and 2% chromosome assignment discrepancy between the HS and LN54 maps. Altogether we positioned 2,126 finished clones on the meiotic and RH maps. Among these clones 23.6% are on the MGH map, 55.1% are on the HS map, 70.0% are on the T51 map, and 47.3% are on the LN54 map. These are well-distributed across the zebrafish genome and encompass a total of 269,812 SNPs, 35,040 insertion-deletions, and 8,732 SSLPs. The mean clone density was one clone per 4.6 cM on the MGH map, 2.9 cM on the HS map, 12.8 cR on the LN54 map, and 71.6 cR on the T51 map. We observe that 93.1% of the genetically positioned BACs have concordant linkage group and Zv6 chromosomal assignment. As an example, Figure 2 shows positioned BACs of each of the four genetic maps and the Zv6 assembly for linkage group 7. An average of 127 candidate SNPs and 4 SSLPs are found on each of the positioned finished BAC clones. The great redundancy of SNPs at each genetic position suggests that the derived SNP maps will be instrumental for mapping in diverse experimental crosses. Additional data file 3 (in five parts) provides the candidate polymorphisms anchored to each genetic map and their positions.

### SNP genetic maps confirm *trilobite *chromosomal localization

**Figure 3:**
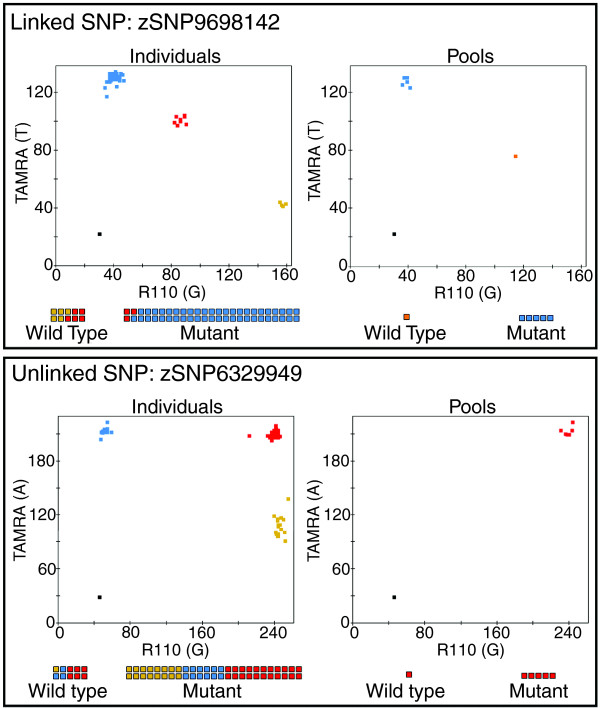
Bulked segregant analysis of the *trilobite *locus employing anchored SNPs. Scatter plots provide SNP genotype data for a linked SNP (upper panel) and an unlinked SNP (lower panel). Data of individual embryo DNA is presented to the left and data of DNA pools is presented to the right. Under each plot is a further schematic representation of DNA sample genotype: 10 wild-type embryos, 50 mutant embryos, 1 wild-type embryo DNA pool, or 5 mutant embryo DNA pools. Homozygotes are shown in yellow or blue, heterozygotes are in red, and negative controls are in black. The data point of the wild-type pool at the linked SNP (top right) is intermediate between the position of a G/T heterozygote and G/G homozygote. Plot axes are in milli-polarization units, indicating relative dye terminator incorporation with single nucleotide primer extension detected by fluorescence polarization.

To test the utility of the novel SNP maps for linkage analysis we investigated the previously cloned zebrafish *trilobite *mutation. The recessive *trilobite *gastrulation mutant was induced by N-ethyl N-nitrosourea mutagenesis in the AB strain and was positionally cloned [8]. The trait is attributed to mutations in the *vangl2 *gene located on linkage group 7, linked to SSLP marker z17411 at 8.9 cM on the MGH genetic map. The SSLP is on chromosome 7 of the Zv6 draft physical assembly. However, part of the *vangl2 *gene is on chromosome 2 of the draft assembly and the remainder is on an unassembled scaffold. We employed SNPs anchored to genetic maps of linkage group 7 (Figure 2) to demonstrate linkage of *trilobite *to its correct genetic position. We selected the closest genetically positioned BAC as zC258D20, which is marked by EST AI544871. This EST is positioned at 18.4 cR on LG 7 of the LN54 RH map, which corresponds to 8.95 cM on the ZFIN composite map. The BAC contains numerous polymorphisms detected in our study and is near the linked SSLP. Embryos resulting from an F1 cross of *tri*^*m*209 ^carriers were evaluated in bulked segregant analysis with SNPs of this BAC. Of 27 tested candidate polymorphisms, 22 (81%) were informative and equivalently distinguished the two parental haplotypes at the locus. Five different mutant pools, each composed of DNA from ten mutant embryos, appeared homozygous at the linked SNPs. In contrast, the genotype of a DNA pool of ten wild-type embryos was an intermediate between a heterozygote and the opposite homozygote at each linked SNP (for example, linked zSNP9698142 (ss48784923); Figure 3). Mutant and wild-type pool genotypes were indistinguishable at an unlinked marker and appeared heterozygous (unlinked zSNP6329949 (ss48401042); Figure 3). Genotyping of individual embryos confirmed the pool results, and yielded a LOD score of 23.95 (θ = 0.03) at the linked SNP. Of 50 mutant embryos genotyped at the linked SNP, 47 were homozygotes (T/T) and three were heterozygotes (G/T), providing an estimated genetic distance of 3 cM between the marker and the *trilobite *locus. In contrast, no wild-type embryos were homozygous for the mutation-linked allele. These results support the effectiveness of the anchored SNP genetic maps for successful application in bulked segregant mapping in zebrafish.

## Discussion

The principal goal of this study was to identify SNPs of finished zebrafish sequence and to position them on current genetic maps as a resource for SNP-based linkage mapping in zebrafish. We identified 548,508 candidate bi-allelic SNPs in zebrafish and demonstrated correlation of their rate of validation with an assigned confidence score for each polymorphism. This rate increases from 71% for SNPs with a confidence score ≥4 to 86% for SNPs with a score ≥7. We also identified 78,327 insertion-deletion polymorphisms and 17,903 SSLPs. We anchored 269,812 of the candidate SNPs to 2,126 loci on 4 principal zebrafish genetic maps (MGH, HS, T51, and LN54). Each of the loci positioned in our study harbor an average of 127 candidate SNPs (and 4 SSLPs) to provide an informative marker in any given cross. These loci are distributed across four genetic maps; the density of the composite SNP-based resource exceeds that of each individual map. Future genetic positioning of the remaining 52% of the 4,410 BACs that harbor candidate SNPs will improve current map coverage. Consolidation to a single reference genetic or physical map and advance characterization of allele frequency in common mapping strains would also aid positional cloning in zebrafish.

To explore the utility of the new resource, we confirmed the known location of the *trilobite *locus through bulked segregant analysis using SNPs anchored to these genetic maps. In our test of SNPs near the *trilobite *locus (the *vangl2 *gene), the relative dosage of A and B allele frequencies of a given SNP within mutant and wild-type DNA pools clearly distinguished the linked and unlinked markers. For a 10 cM map, a SNP will be no more than 5 cM from the trait locus, thus a mutant pool may harbor 5% (1 in 20) recombinant chromosomes, dependent upon sampling. Several mutant pools tested did harbor 1 in 20 recombinant chromosomes in the vicinity of the linked marker. In our genotyping assays of SNPs on the linked BAC, mutant pools with a recombinant chromosome were, at most, only slightly distinguishable from those mutant pools without a recombinant chromosome, while remaining easily distinguishable from wild-type pools. These results suggest that an informative 10 cM map is sufficient for bulked segregant mapping employing SNPs.

Genetic diversity of the common zebrafish mapping strains is relatively uncharacterized. The identified SNPs illustrate that the TU strain is not inbred for a large portion of its genome and is likely to be a laboratory founder population. Sequence evidence of polymorphism for 98.8% of candidate SNPs with a confidence score ≥4 is restricted to TU data. However, we observe extensive polymorphism in other strains for these SNPs, potentially marking haplotypes of a common ancestral origin. Many of the validated SNPs were informative within and across the three tested partially inbred lines (with origins in the AB and DAR strains) [9,17,18], as well as within the AB-derived *trilobite *mapping cross [8]. It should be possible to use this SNP resource both for linkage mapping in the common laboratory strains, and to better characterize the genetic diversity and lineage history of the various mapping strains. One approach to accomplish this is through analysis of SNP haplotypes in regions of high linkage disequilibrium sampled across the genome. This knowledge would in turn enable the selection of divergent strains for experimental crosses, maximizing information content of sampled polymorphisms. SNP haplotype diversity of common zebrafish strains may further be of interest for mapping modifier loci in quantitative traits. It is also conceivable that dense SNP maps could enable relatively rapid creation of inbred strains by sequential selective crosses that most efficiently maximize homozygosity.

Many zebrafish traits have been produced in large-scale mutagenesis screens, and most remain to be positionally cloned. Current SNP genotyping technology is capable of bulked segregant mapping of large numbers of these traits concurrently. Subsequent fine mapping of recombinant progeny in the vicinity of each trait locus may be accomplished with additional regional markers, ideally with advance knowledge of marker information content in common mapping strains. Accomplishing these tasks using SNPs anchored to accurate genetic or physical maps has the potential to be less labor-intensive, faster, and more precise than traditional mapping employing SSLPs. The density of SNPs in the zebrafish genome greatly exceeds that of SSLPs, offering a higher potential mapping resolution. We observed one SNP per 1.8 kb and one SSLP per 40 kb of finished clone sequence. Genotyping of SNP markers may be accomplished using standard manual gel electrophoresis-based methods such as single-stranded conformation polymorphism, restriction fragment length polymorphism, or direct sequencing. However, robust automated commercial SNP genotyping systems are capable of higher throughput at a lower cost. Continued development of accurate SNP-based genetic maps will empower disease gene identification and complex trait mapping employing the zebrafish model organism.

## Conclusion

This study identifies a dense polymorphism resource for genetic mapping in the zebrafish model organism, including SNPs, SSLPs, and insertion-deletions. Intriguingly, we observe that the neighboring sequence context of zebrafish SNPs is non-random. Half of the newly discovered polymorphisms are anchored to genetic maps as well as to the current draft physical map to improve utility for positional cloning. We illustrate an efficient bulked segregant mapping strategy using the resource to rapidly identify linkage through assay of only two samples: a wild-type and a mutant DNA pool. Dense polymorphism maps and high-throughput genotyping technologies will accelerate genetic mapping in a powerful vertebrate model organism that has proven instrumental in understanding human development and disease.

## Materials and methods

### Repeat database creation

The Zv5 genome assembly was downloaded from ENSEMBL [19]. The genome was masked for simple sequence repeats (using the simple sequence database from RepBase [20] and NCBI's BLAST, v2.2.10 [21]). Genomic scaffolds were ordered according to decreasing size. An initial scaffold size threshold was chosen to allow for more rapid initial BLAST runs than could have been realized from larger scaffolds. Those ≤200 kb were selected and compared (in order of decreasing size) against the entire genome using BLAST to find repeated regions (word size = 40, e-value = 1 × 10^-100^, filter = false, number of alignments = 1,000,000). The comparisons were allowed to run until approximately 3GB of result files were generated. These results were then used to generate a FASTA database of repeated sequence. These repeats were then located on each genomic scaffold by BLAST (W = 30, e = 1 × 10^-35^). Found repetitive sequences were masked on each scaffold. The masked scaffolds were then used for comparison against the entire genome, as above, until another approximately 3GB of result files were generated. This process looped until every scaffold had been compared against the genome, with the repeated sequences found in each round being added to the previously created database. Once all scaffolds of 200 kb or less were compared, those larger than 200 kb were processed for completion of the repeat database. In total, 56% of the Zv5 assembly and 36% of finished clones were observed to be non-unique by these criteria.

### SNP discovery

Clone sequence and whole genome shotgun sequence produced by the *Danio rerio *Sequencing Group at the Sanger Institute was obtained from online databases [22,23]. These were used to create a clone sequence FASTA database and a shotgun sequence FASTA database. Shotgun sequence was from the TU strain; 97.4% of finished sequence was from the TU strain, and 2.6% was from the AB strain. The finished clones were masked using the RepBase simple sequence database and the custom zebrafish repeat database (W = 30, e = 1 × 10^-35^). Each masked finished clone was then compared via BLAST to independent unfinished clones (W = 50, e = 1 × 10^-100^), and to shotgun sequence (W = 50, e = 1 × 10^-125^). Results of the BLAST runs were examined to find sequence mismatches, which were stored in a database. Where multiple adjacent base deletions (or insertions) were observed, results consistent with a single insertion-deletion record were summarized. Each variant was then given a confidence score to highlight those candidate SNPs with only two alleles that were seen in similar proportions, and at sequence regions sampled with greater frequency. This score was calculated as follows: *conf_score *= (*minor_allele_count*/*major_allele_count*)/(*ceiling*(*other_allele_count*/*2*))^2 ^× (*minor_allele_count + major_allele_count*). The *ceiling(x) *function gives the smallest integer ≥*x*. Base quality scores were not employed given available computational resources, and given that simple sequencing errors received low study-assigned confidence scores. We directly correlated study-assigned confidence score with experimental evidence of validation rather than compare to alternative SNP prediction methods.

Where a region of the genome is repetitive but represented uniquely in a draft assembly, our repeat database will be insufficient to mask sequences in the process of SNP discovery. With discovery of candidate polymorphisms experimentally consistent with non-unique loci, the scoring algorithm was further modified. If the candidate SNP had a sequence depth of >25, the confidence score was penalized (*conf_score*/(*minor_allele_count + major_allele_count*)) to exclude potential non-unique sequences. The depth of 25 was empirically chosen based upon available experimental evidence during SNP validation. Candidate SNPs with multiple alignments on an unassembled clone or multiple alignments on a single shotgun sequence (sequence not used for repeat database creation) were assigned a confidence score of zero. The mean sequence depth for a SNP with a confidence score ≥4 was 9.73-fold.

### SSLP discovery

Finished clones were analyzed to find simple tandem repeats with repeat units of between 2 bp (dimers) and 10 bp (decamers), and at least 6 repeats. A custom algorithm scanned the target sequence for a given repeat type and paused with two occurrences. Next it filtered out repeats composed of monomers. The bases following each repeat were then examined for continuation of the repeat unit. If the number of repeat units exceeded six, then the discovered repeat was reported. Concatenated repeats were identified by examining the list of reported repeats, sorting by location, and assessing whether the end of one repeat was less than one repeat unit from the start of an adjacent repeat. Simple tandem repeats residing in non-unique sequence were omitted from further consideration. Each simple tandem repeat with a total of 400 bp of flanking sequence was then compared to the previously created clone and shotgun sequence databases using BLAST (W = 50, e = 1 × 10^-90^). These sequences were then examined to calculate the length of each instance of the SSLP.

### Assignment of clone positions to genetic maps

In order to assign BACs (and SNPs anchored to them) to positions in existing genetic maps, we identified annotated markers of these genetic maps contained in the sequenced BACs. Annotations on meiotic (HS and MGH) and RH (T51 and LN54) maps were downloaded from ZFIN [24]. Additional ESTs genetically mapped by Woods *et al*. [13] and not present in the ZFIN database were included. Zv6 sequence assembly positions for SSLPs, SNPs, genes, ESTs, mRNAs, and cDNAs were downloaded from ENSEMBL [25,26] and from UCSC Genome Bioinformatics [27]. Annotations that were not positioned by these data sources were positioned on the Zv6 assembly using BLAST (W = 40, e = 1 × 10^-70^). Annotations with a unique position, >70% sequence alignment and >95% sequence identity by BLAST were assigned genomic positions. In total, 619 SSLPs, 500 SNPs, 2,004 genes, 132 cDNAs, and 7,100 ESTs that were annotated on the HS, MGH, T51, and LN54 maps were located on finished clones employed for polymorphism discovery.

### Fish stocks

Frozen TU population fish (20 adult males and 20 adult females) were kindly provided by Dr Zoltan Varga and staff of the Zebrafish International Resource Center (University of Oregon, Eugene, OR, USA). They were offspring from 25 or more group matings of TU wild-type fish. Each group mating consisted of two females and three males. Two frozen adult male fish of each of the partially inbred strains SJD (derived from the Darjeeling line), SJC (derived from C32), and SJA (derived from AB) were kindly provided by Dr Stephen Johnson (Washington University, St Louis, MO, USA). Genomic DNA was extracted using the PureGene Genomic DNA Purification Kit (Gentra Systems, Minneapolis, MN, USA). DNA was quantified by PicoGreen assay (Molecular Probes/Invitrogen, Carlsbad, CA, USA).

### Genotyping

We randomly selected 392 candidate SNPs, each with a confidence score ≥4, for assessment of polymorphism within the TU population and partially inbred lines (SJD, SJC, and SJA). We genotyped SNPs by single nucleotide primer extension and fluorescence polarization in 384-well format [28]. Reaction processing entailed three steps: a 4.4 μl PCR reaction, addition of 4 μl of an exonuclease I (New England Biolabs, Beverly, MA, USA) and calf intestinal alkaline phosphatase (Promega, Madison, WI, USA) reagent mix to degrade unincorporated primer and dephosphorylate dNTPs, and a final addition of 4 μl of an Acyclopol and Acycloterminator reagent mix for the primer extension reaction (AcycloPrime™ FP SNP Detection System, Perkin-Elmer, Boston, MA, USA). Each PCR mixture included 0.1 unit AmpliTaq Gold DNA polymerase, 1× Buffer II (Applied Biosystems, Foster City, CA, USA), 2.5 mM MgCl_2_, 0.25 mM dNTPs, 335 nM of each primer, and 2 ng DNA template. We detected incorporation of R110- and TAMRA-labeled terminators by fluorescence polarization on a Molecular Devices/LJL Analyst HT.

All assays were conducted using both forward and reverse strand extension primers: both assays worked for 304 (77.6%), 1 of the 2 assays worked for 71 (18.1%), and both assays failed for 17 of 392 candidate SNPs (4.3%). In total, 375 yielded an informative assay, an overall conversion rate of 95.7%; for these 375 SNPs we obtained 95.8% of genotypes sought among the 46 screened DNAs. The concordance rate among paired forward and reverse strand genotypes of unique loci was 99.6%. Amplimer and extension primer sequences are provided in Additional data file 4. Primer design was accomplished using custom software to minimize primer-dimer and hairpin loop artifacts, to avoid non-unique sequence and variant sites, and to target a Tm of 56°C (average length of 20 bases).

### Linkage of *trilobite*

We designed fluorescence polarization assays for 27 candidate SNPs from BAC zC258D20 that is positioned on linkage group 7 near the *tri *locus, zSNPs 9697125, 9697126, 9697739, 9697740, 9697743, 9698041, 9698042, 9698043, 9698044, 9698045, 9698049, 9698050, 9698051, 9698140, 9698141, 9698142, 9698151, 9698155, 9698159, 9698161, 9698162, 9698163, 9698166, 9698167, 9698309, 9698311, and 9698312. The SNPs were assayed in DNA from 50 *tri *mutant and 10 wild-type progeny of a cross of F1 wild-type *tri*^*m*209 ^carriers. Five equal-part DNA pools of ten mutant embryos each, and one pool of ten wild-type embryos were used for bulked segregant analysis. Individual mutant embryos were genotyped to assess the genetic distance between the markers and the trait locus. An unlinked SNP of linkage group 10 (zSNP6329949) was additionally genotyped for data comparison. Amplimer and extension primer sequences are provided in Additional data file 4.

### Data availability

Zebrafish polymorphism data have been submitted to dbSNP [10] [NCBI:ss48400959 to NCBI:ss49840082], to ZFIN [24], and to zBase [29]. These include all SNPs and indels of confidence score ≥4, as well as SSLPs.

## Additional data files

The following additional data are available with the online version of this paper. Additional data file 1 is a figure that presents allele frequency diagrams of genotyped SNPs. SNP positions within the Zv6 sequence assembly (chromosome-base number) are provided. Where not present in the Zv6 assembly, a single asterisk indicates SNP position within the Zv5 assembly or a double asterisk indicates SNP position within the Zv4 assembly. Additional data file 2 is a table providing the proportions of single nucleotide variants observed in *D. rerio *sequence, categorized by assigned confidence score and by substitution type. Additional data file 3 is a five-part table (a-e) in text file format amenable to database import that provides information for genetically positioned candidate zebrafish SNPs. Additional data file 4 is a table of designed genotyping assays for validated SNPs. Additional data file 5 is a large FASTA format file containing Zv5 repetitive sequences.

## Supplementary Material

Additional data file 1 SNP positions within the Zv6 sequence assembly (chromosome-base number) are provided. Where not present in the Zv6 assembly, a single asterisk indicates SNP position within the Zv5 assembly or a double asterisk indicates SNP position within the Zv4 assembly. Click here for file

Additional data file 2 Proportions of single nucleotide variants observed in *D. rerio *sequence, categorized by assigned confidence score and by substitution type Click here for file

Additional data file 3 A five-part table (a-e) in text file format amenable to database import Click here for file

Additional data file 4 Designed genotyping assays for validated SNPs Click here for file

Additional data file 5 Zv5 repetitive sequences Click here for file

## Figures and Tables

**Table 1:** Adjacent base context of unique finished sequence and candidate SNP sites in *D. Rerio*

Flank motif	DNA sites	C/T SNPs	A/G SNPs	A/T SNPs	G/T SNPs	A/C SNPs	C/G SNPs
^5'^T-N-A^3'^	10.1%	15.2%	15.1%	16.5%	13.4%	13.3%	10.7%
A-N-A	11.9%	9.8%	9.7%	13.0%	7.9%	19.9%	12.8%
T-N-T	11.9%	9.5%	9.9%	12.9%	19.8%	7.8%	12.8%
A-N-T	9.9%	7.4%	7.5%	13.0%	8.5%	8.4%	9.9%
A-N-G	5.3%	7.4%	5.7%	4.6%	5.4%	4.1%	3.9%
T-N-C	5.5%	6.0%	4.9%	3.7%	4.7%	4.2%	7.1%
T-N-G	4.9%	5.9%	5.6%	5.1%	7.4%	2.4%	3.5%
C-N-T	5.3%	5.7%	7.2%	4.9%	4.2%	5.4%	3.3%
C-N-A	4.9%	5.5%	5.9%	5.1%	2.4%	7.4%	3.6%
G-N-A	5.5%	4.9%	6.0%	3.6%	4.1%	4.8%	7.3%
G-N-G	3.5%	4.6%	2.9%	2.6%	4.7%	1.9%	2.6%
G-N-T	5.3%	4.6%	3.7%	3.1%	6.6%	3.2%	6.4%
C-N-G	3.9%	4.4%	4.3%	4.8%	3.7%	3.5%	1.9%
A-N-C	5.3%	3.6%	4.6%	3.1%	3.4%	6.7%	6.1%
C-N-C	3.5%	2.9%	4.5%	2.7%	1.7%	4.9%	2.8%
G-N-C	3.2%	2.7%	2.6%	1.5%	2.2%	2.1%	5.2%
	*N *= 457,748,448	*N *= 141,416	*N *= 141,168	*N *= 111,434	*N *= 62,017	*N *= 62,220	*N *= 30,253

**Table 2:** Ranks of adjacent nucleotide proportions of candidate SNPs in *D. Rerio*

	Observed proportion		Proportion bias	
		
SNP type	-1	+1	-1	+1
All	T » A » C > G*	A » T » G > C	T > C > a ≈ g^†^	A > G > t ≈ c
Transition	T » A » C > G	A » T » G > C	T > C > g > a	A ≈ G > c > t
Transversion	T > A » C > G	A > T » G > C	T > A > c > g	A > T > g > c
A/G	T » A » C » G	A » T » G > C	C > T » g > a	A > G > c > t
C/T	T » A » C > G	A » T > G » C	T > C > g > a	G > A » c > t
A/C	A » T » C » G	A » T » C » G	A > C » t ≈ g	A » C » g > t
G/T	T » A » G » C	T » A » G » C	T » G » c > a	T > G » a > c
A/T	T > A » C » G	A > T » G » C	T > A > c » g	A > T > g » c
C/G	T > A » G » C	A > T » C » G	G > T > A » c	C > A > T » g

*» denotes greater than 5% difference between two nucleotide proportions, > 1%-5% difference, and ≈ less than 1% difference. ^†^A lower-case letter denotes a negative bias of the observed nucleotide proportion compared with the genome average.
